# Structural characterisation and degradation of Mg–Li thin films for biodegradable implants

**DOI:** 10.1038/s41598-023-39493-9

**Published:** 2023-08-03

**Authors:** Lisa Hanke, Lea K. Jessen, Felix Weisheit, Krathika Bhat, Ulrike Westernströer, Dieter Garbe-Schönberg, Regine Willumeit-Römer, Eckhard Quandt

**Affiliations:** 1 Inorganic Functional Materials, Institute for Materials Science, Faculty of Engineering, Kiel University, Kiel, Germany; 2 Institute of Metallic Biomaterials, Helmholtz Centre Hereon, Geesthacht, Germany; 3 Marine Climate Research, Institute of Geosciences, Faculty of Mathematics and Natural Sciences, Kiel University, Kiel, Germany

**Keywords:** Biomaterials, Materials for devices, Corrosion

## Abstract

Freestanding thin films of Mg–Li (magnesium–lithium) alloys with a Li mass fraction between 1.6% (m/m) and 9.5% (m/m) were prepared and studied with respect to their structure and degradation properties. With increasing Li content, the microstructure deviates from hexagonal Mg–Li with strict columnar growth and preferred orientation, and additional cubic Mg–Li and Li_2_CO_3_ occur. The corrosion rate was measured in Hanks’ balanced salt solution by potentiodynamic polarisation and weight loss measurements to investigate biodegradation. Influences of the orientation, phase and protective layer formation lead to an increase in corrosion from 1.6 to 5.5% (m/m) from 0.13 ± 0.03 to 0.67 ± 0.29 mm/year when measured by potentiodynamic polarisation but a similar corrosion rate for 9.5% (m/m) and 3% (m/m) of Li of 0.27 ± 0.07 mm/year and 0.26 ± 0.05 mm/year.

## Introduction

Magnesium and its alloys are widely studied as materials for applications in the medical field due to their biodegradability. Different elements such as, e.g., Ca, Zn or rare earth elements (REE) are included to improve mechanical properties or tailor the degradation rate to adjust them for applications as, e.g., stents or bone implants^1–4^. Additional to the advantage of having an implant which degrades after it is no longer required, the possible therapeutic effects of the implants are explored by, e.g., loading stents with drug-eluting layers^5^ or using the corrosion process and changes in the environment such as pH and hydrogen evolution directly for its antibacterial properties^6^.

In line with the idea to use the implant itself as treatment, an alloy including the therapeutically active element lithium will be analysed in this study. Lithium is used in treatments for mood disorders, in particular bipolar disorder, and is also studied to have effects on Alzheimer’s and Parkinson’s disease^7–10^. Magnesium itself shows also neurological effects^11^. Therefore, the degradation and, thus, continuous release of both the magnesium and additional elements would allow a local treatment in the brain. If a controlled and local release is achieved by understanding the degradation of the material, side effects which can occur during the treatment with Li^12^ could be reduced.

**Figure 1:**
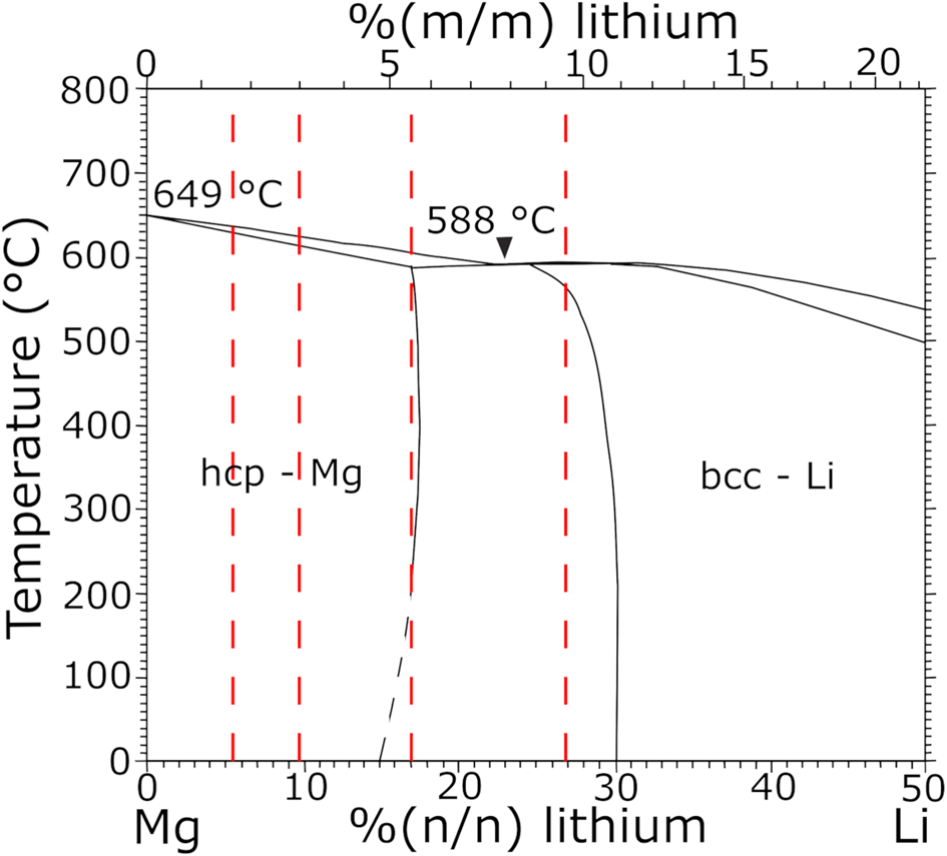
Mg–Li phase diagram, adapted from^13^. The region with a Li fraction from 0 to 50% (n/n) is depicted and the concentration of sample films (Li mass fraction of 1.6% (m/m), 3% (m/m), 5.5% (m/m) and 9.5% (m/m)) are marked. Low Li concentrations lead to a Mg rich α-phase with a hcp structure and high Li concentrations to a β-phase with a bcc structure. In the mixed phase regions, α-phase and β-phase are formed.

For Mg–Li, the structure in bulk materials differs from pure Mg by a reduction of the distance in *c*-direction and a phase change to a body centred cubic (bcc) phase (β phase) for higher Li fractions (Mg–Li phase diagram, Fig. 1^13^). This change leads to additional non-basal slip on the prismatic planes, twinning, and more ductile properties even in hcp Mg–Li alloys. For higher Li fractions, the addition of the second phase can significantly change the mechanical properties. Li et al. showed that cracks are preferably formed at the phase boundaries which is facilitated by the difference in the number of gliding systems present in both phases and, thus, a difference in stress accumulation^14^. Additionally, the ageing of the second phase and change from bcc to hcp phase even at room temperature influences the properties over time^14,15^.

The corrosion rate of Mg–Li alloys is influenced by several factors such as the high activity of Li, change of microstructure and surface films. The low electrochemical potential of Li leads to an increase in the cathodic kinetics and a more significant shift of the pH. Filiform corrosion is found to be one of the main corrosion processes occurring for Mg–Li alloys in the α or α + β materials^16–18^. For films with mixed phases, micro-galvanic coupling is found as a main factor for an increase in corrosion rate with preferred corrosion and pitting at the phase boundaries^19,20^. However, the diversity of microstructure and protective layers formed during corrosion makes a clear indication of the influence of the different factors on the corrosion rate difficult. Li et al. showed that the corrosion rate decreases from α + β > α > β^18^. The lower corrosion rate of the bcc phase is assumed to be due to the high density and stability of a formed protective layer. While for the Mg rich phase, mainly a porous Mg(OH)_2_ layer is expected to form during corrosion^21,22^, the layer structures of surface films formed on Mg–Li including the bcc phase are complex. Xu et al., e.g., analysed the structure of the natural film formed in air as a Li_2_CO_3_ film on the surface, Mg oxide and Li oxide film underneath and Mg-rich film before the bulk material^23^. Other studies claim for films formed in air or during corrosion several compounds including carbonates, oxides and hydroxides of both Li and Mg, often separated in a layer structure^16,24,25^. Previous studies assumed or suspected that the formed Li_2_CO_3_ has the main influence on the higher corrosion resistance of the bcc phase^23,26,27^. The Pilling–Bedworth ratio (PBR), which is a measure for film stress and, thus, identifies a stable film for 1 < PBR < 2, is > 1 for all Mg:Li ratios for Li_2_CO_3_. Thus, it could already be formed for lower Li mass fractions in the hcp phase^18^. Yan et al. suggest another possible influence as Li doping and therefore strengthening the MgO and hindering the formation of the more porous and less protective magnesium hydroxide. Since the critical Li fraction for forming a stable layer of MgO is calculated to be around 15–18 at. % (4.8–5.9%(m/m)), this is in agreement with the formation of the layer only on Mg–Li with β or α + β^28^. Therefore, with the assumption of the formation of a stable MgO layer by Li doping, the higher Li fractions leads to a decrease in corrosion rate by changing the film stress.

However, it has to be noted that the corrosion of Mg–Li with different phases is a complex system, leading to other studies showing the lowest corrosion rate for the hcp phase^19^. The main influences on the actual corrosion rate are therefore not just Li content and phases but also the microstructure of the material.

For neurological implants, small freestanding films and structures in the sizes of a few µm–mm are needed. Previous to this paper, no extensive studies of Mg–Li thin films regarding the growth and properties are available to our knowledge. As found for thin films of other Mg alloys, significant influences on the properties in comparison to bulk materials are expected. For different Mg alloys such as Mg–Ag or Mg–REE, the structure of thin films was studied^29–33^. For sputter-deposited films, the hexagonal close packed (hcp) magnesium phase (α phase) is strongly textured with a preferred growth direction of [001] and a columnar grain structure is apparent^31,33–35^. The strong texture influences the deformation by, e.g., reducing work hardening and influences therefore the mechanical properties^33^. The corrosion of thin films in comparison to bulk is shown to be more homogeneous with less effect of pitting^35,36^. Additionally, the corrosion resistance and oxidation of different planes are different due to the packing and binding energies, thus, the corrosion rate is influenced by the texturing of the films^37^. The (001) plane is found to show the lowest corrosion rate due to the densest packing, however, since the faster oxidation of other planes could lead to a protected surface, the actual influence on the corrosion rate cannot be directly predicted^38,39^. Since the properties are highly dependent on structure and microstructure, they can be influenced for the same alloy by changing the sputtering parameters, leading to higher densities or differences in the film growth depending on the energy available for diffusion of the atoms^29,32,40–42^.

In this study, Mg–Li thin films with a pure hcp or an α + β structure (Mg–Li phase diagram, Fig. 1) are prepared via magnetron sputtering. To gain insight into the corrosion process dependent on the specific structure present in the thin films, studies with respect to their growth and microstructure are carried out to allow a correlation with influences on the corrosion rate. Additionally, the possibilities to influence and tune those to meet specific requirements given for applications are discussed.

## Results

### Composition and microstructure

The compositions of the prepared Mg–Li thin films are marked with red dashed lines at mass fractions of 1.6% (m/m), 3% (m/m), 5.5% (m/m) and 9.5% (m/m) in the phase diagram in Fig. 1 (fractions given in Supplementary Table 1). As shown, two sample types are prepared with Li mass fractions leading to pure hexagonal phase, while the other two theoretically consist of α and β phase with approximately 89% (Mg–5.5Li) or 22% (Mg–9.5Li) α phase.

**Figure 2:**
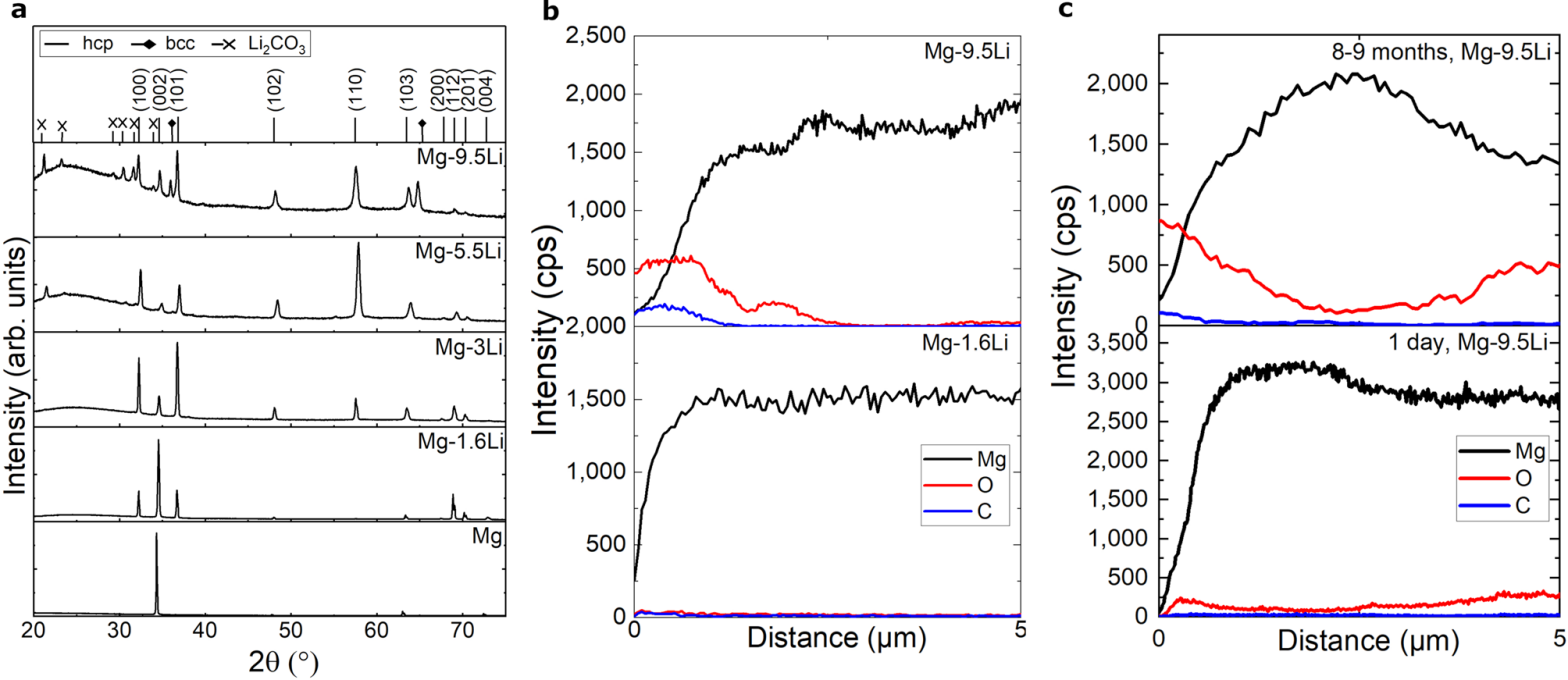
Structure and composition of freestanding thin films (**a**) XRD diffractograms for Mg, Mg–1.6Li, Mg–3Li, Mg–5.5Li, Mg–9.5Li thin films. The positions of the hcp and bcc Mg–Li phases and Li_2_CO_3_ are marked. Additionally, the orientation of the corresponding planes for the hcp phase are indicated, (**b**) EDX line scans of cross sections of Mg–1.6Li and Mg–9.5Li thin films as used for corrosion measurements, (**c**) EDX line scans of cross sections of Mg–9.5Li freestanding thin films after 1 day and 8–9 months.

Very low iron contaminations are measured for all film compositions. Representative XRD diffractograms for all compositions are displayed in Fig. 2a in comparison to pure Mg prepared by the same procedure. While pure Mg exhibits a strong texture with a main peak of (002) at 34.3°, including 1.6% (m/m) Li leads to less preferred growth and for Mg–3Li, a random orientation is identified (Supplementary Fig. 1). For both materials in the α + β phase, the hexagonal phase shows a preferred orientation of (110). Additional bcc can be identified as small peaks in the diffractogram, and additional studies of the reciprocal space allow the identification of strong (110) peaks for Mg–9.5Li at an angle of χ = 32°–40°, indicating a strongly textured β-phase. While the lattice parameter *a* is only slightly decreased from Mg–1.6Li to higher Li mass fractions, *c* is reduced, especially for the increase of Li from 1.6% (m/m) to 3% (m/m) (peak shift in Fig. 2a, calculated lattice parameters in Supplementary Table 2). When the second phase is present, the parameters do not decrease further since the added Li is included in the additional phase. Of interest are as well the peaks at, e.g., 21.3°, 23.3°, 29.4° and 34.1° for Mg–5.5Li and Mg–9.5Li which indicate the existence of Li_2_CO_3_ (Supplementary Table 3). Because of the formation of this additional phase, the amount of β-phase is reduced.

**Figure 3:**
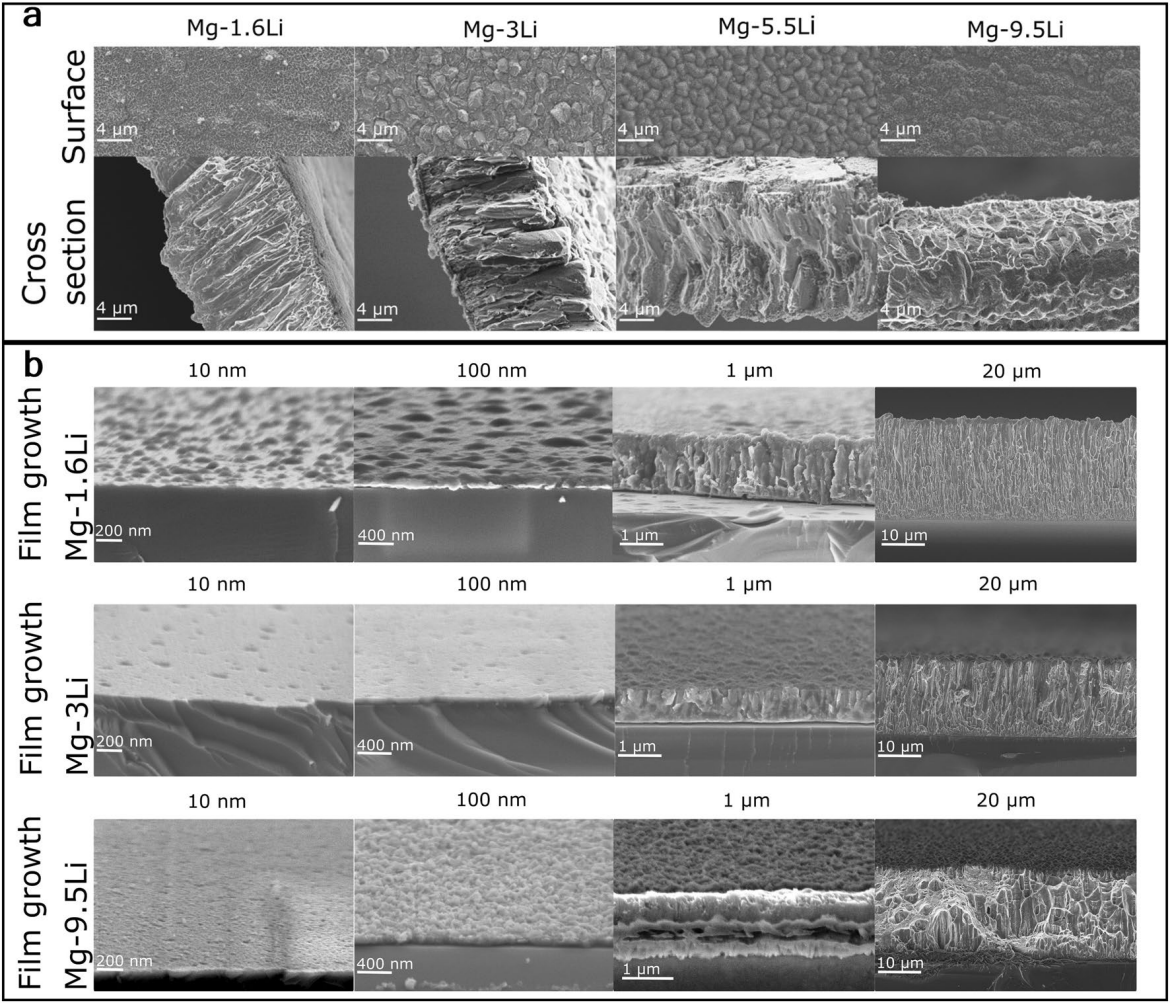
SEM images (**a**) Surface and cross-section of freestanding Mg–Li thin films. (**b**) Side view or cross-section of Mg–Li films (Li: 1.6% (m/m), 3% (m/m), 9.5% (m/m)) on Si substrate with thicknesses of 10 nm, 100 nm, 1 µm and 20 µm.

Investigations via EDX show a layer including carbon and oxygen on the surface of Mg–9.5Li, thus, the Li_2_CO_3_ identified by XRD can mainly be assigned to a film formed on the surface of the samples when those are stored in air. The formation of Li_2_CO_3_ in humid air for β-phase Li is also shown in previous studies^23,28^. A layer of Li_2_O is formed on the surface of alloys with high Li content and can react further to Li_2_CO_3_ if CO_2_ is present in the surrounding atmosphere^23^. There is no significant compositional change over the layer thickness for, e.g., Mg–1.6Li (Fig. 2b). Cross-sectional images of the different Mg–Li alloy freestanding thin films with a thickness of 10 µm are given in Fig. 3a for analysis of the microstructure. Mg–1.6Li exhibits a columnar growth with a constant diameter of approximately 500 nm to 1 µm over the whole film thickness. This structure is also identified for pure Mg films with the strong (001) texture prepared via magnetron sputtering^30^. For Mg–3Li, smaller grains are formed close to the substrate while columns start after a few 100 nm with increasing diameter up to 1.5 µm to 2 µm with a few columns exhibiting a diameter of around 4 µm. Less columnar growth is visible for Mg–5.5Li and cannot be identified for the highest Li mass fraction. The surface, however, still exhibits a structure which leads to the identification of grain sizes of approximately 1.5–2 µm. The difference in the cross-sectional images can not only be assigned to a change of columns to a different microstructure because of additional phases but also to less preferred fracture at the grain boundaries during bending. This is influenced by, e.g., voids formed due to the self-shadowing of the columns. For Mg–9.5Li, oxidation of the samples also plays a major role in the visible structure since the samples are highly affected. While a thin oxide film is formed for all films and is apparent in the surface images in Fig. 3a, only for films with higher Li content (Mg–9.5Li) the oxide grows significantly until the film is completely oxidized (Fig. 2c).

To classify the microstructures further, they can be compared with the structure-zone model^41,43^. The substrate temperature during the deposition was (49–54) °C for Mg–1.6Li, (54–66) °C for Mg–3Li and (60–66) °C for Mg–5.5Li and Mg–9.5Li samples. This leads to *T*/*T*_m_ = 0.36 for Mg–1.6Li and *T*/*T*_m_ = 0.39 for Mg–9.5Li as the highest and lowest possible values, leading in theory to structures in the T-zone, changing into zone 2. For higher zones, the grain boundaries get denser, and the defect density decreases, therefore fewer voids occur which could influence the fracture at grain boundaries. Additionally, a shift from straighter fibres to more complex structures at lower layers for Mg–3Li is in good agreement with the microstructural cross-sections.

### Film growth

To identify and understand the differences in microstructure, the growth of Mg–1.6Li, Mg–3Li and Mg–9.5Li films is additionally analysed. The sputter times were chosen according to the sputtering rates for 10 µm to result in approximately 10 nm, 100 nm, 1 µm and 20 µm. The results are displayed in Fig. 3b.

The growth for the lowest Li mass fraction starts with island growth, similar to pure Mg^44^. The film covers the whole surface for a thickness > 10 nm, resulting in columnar growth. The energy of the particles from the sputtering process itself and the low temperature of the substrate do not allow sufficient diffusion for a more homogeneous growth^45^. The columnar growth is additionally in good accordance with the structure formed with the preferred orientation of (001) because the fastest growth for hexagonal faces is in direction of the c-axis^45^.

The film growth process is studied to be highly influenced by including alloying elements^44^. For Mg–3Li, the layer formation starts with layer growth, including only few defects in a 10 nm thick layer. After approximately (400–500) nm, the growth changes to columnar growth. This change can be attributed to film stress which accumulates over the layer and changes the energetically favourable growth. Pores and voids can already be identified after the column growth for a layer thickness of 1 µm. Even though columnar growth is visible, the homogeneity of the signal on the reciprocal space mapping from XRD shows that a random orientation is formed throughout the whole film (Supplementary Fig. 1). Since the growth is not as strictly orientated as for Mg–1.6Li, it leads to an increase in column diameter and void formation.

For Mg–9.5Li, the film growth at the beginning cannot be directly identified as island growth, however, at a film thickness of approximately 100 nm, a grain-like surface with a high roughness is visible. Even though these samples were measured directly after preparation and the oxidation of the samples is thus minimal, a colour change of the samples showed a slight oxidation even for the fastest possible measurement and therefore influences especially the thinnest layers. For the thick films, samples with only minor oxidation exhibit columnar structures. However, the sample is only partially breaking at the grain boundaries and, thus, the structure is less pronounced.

### Tensile testing

**Figure 4:**
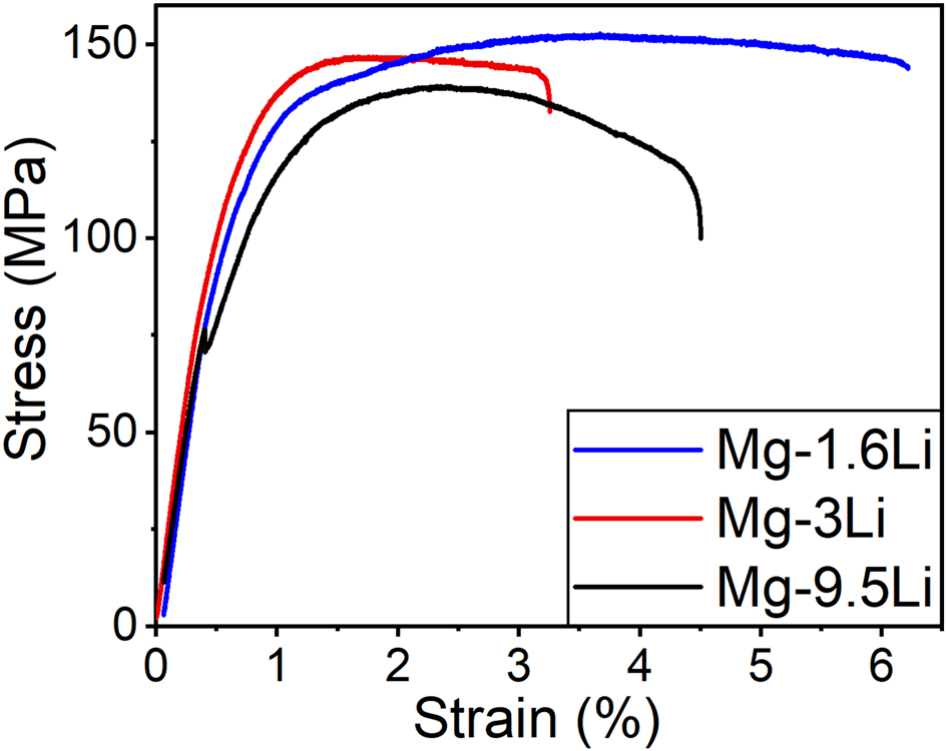
Exemplary stress strain curves of Mg–1.6Li, Mg–3Li and Mg–9.5Li dog-bone shaped thin films.

The mechanical properties were studied by tensile testing perpendicular to the growth direction of the films. Since Mg–5.5Li freestanding thin films are very brittle, possibly partially due to oxidation, no tensile test measurement is possible. Thus, except for the poor mechanical stability, no further analysis of the properties is possible for this sample type. Exemplary stress–strain curves for the other tested Mg–Li alloys are shown in Fig. 4 and the strengths and elongations are listed in Table 1.

**Table 1:** Average values and standard deviation of tensile strength *σ*_max_, yield strength *R*_p0.2_ and elongation at break *ε*_max_ in comparison to pure Mg samples^30^.

Mg–Li alloy	*σ*_max_ (MPa)	*R*_p0.2_ (MPa)	*ε*_max_ (%)
Mg–1.6Li	148 ± 10	110 ± 10	6.1 ± 0.9
Mg–3Li	144 ± 10	116 ± 8	3.0 ± 0.8
Mg–9.5Li	138 ± 10	101 ± 20	3.4 ± 0.9
Mg^30^	171	153 ± 8	7 ± 4

In comparison to pure Mg samples with the same sample shape and measured with the same set-up^30^, adding Li lowers the tensile strength and especially the yield strength. For Mg, the main slip system is basal slip but pyramidal slip is also proposed to be available in a smaller amount due to higher energies^29,30,46^. For the (001) orientated samples, the alignment of the planes in one direction leads to a sharp change from elastic to plastic deformation. Including additional orientations hinders the gliding between grains, however, since not only the orientation but also the lattice structure is influenced by the addition of Li, this does not increase the tensile strength directly but increases the difference between tensile strength and yield strength. While the maximum strain for Mg–1.6Li is similar to pure Mg samples, it is decreased for Mg–3Li and Mg–9.5Li. Examination of the cross-section after tensile testing shows a moderately ductile fracture and no preferred breakage at the grain boundaries, therefore excluding this as a main influencing factor on the fracture mechanism during tensile test, even though Mg–1.6Li exhibits fracture at the grain boundaries during the fracture by bending.

### Corrosion rate

**Figure 5:**
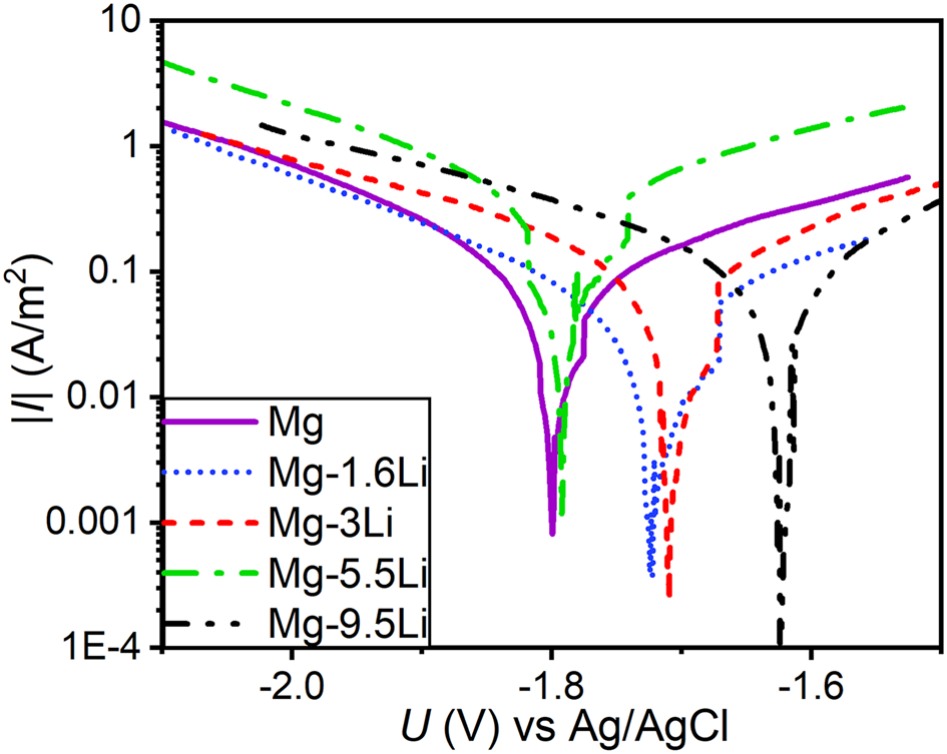
Exemplary Tafel plots obtained by potentiodynamic polarization measurements vs Ag/AgCl reference electrode in Hanks’ balanced salt solution at a pH of 7.4 ± 0.2 and 37 ± 1 °C for Mg, Mg–1.6Li, Mg–3Li, Mg–5.5Li and Mg–9.5Li thin films.

Exemplary potentiodynamic polarisation curves for all studied Mg–Li alloys and Mg are shown in Fig. 5 after 5 min of immersion. For Mg, the intrinsic corrosion rate measured by weight loss measurements in concentrated chlorine solution of highly pure Mg ingots is 0.3 mm/year^47^. Similar values have also been found for high purity Mg in Hanks’ balanced salt solution over long measurement times^48,49^. The corrosion rates of Mg–Li in this study will be compared to Mg thin films sputtered with the same technique and similar purity of the starting target to analyse the influence of Li on the structure and activity, and, thus, the change of corrosion rate. It has to be noted that pure Mg in this case is not actual ultra-high purity Mg which is proven to have a lower corrosion rate than the Mg used here which might include impurities^50^.

Even though Li has a lower electrochemical potential than Mg thin films prepared by the same process^31^, the corrosion potential *E*_corr_ is slightly increased for Mg–1.6Li, Mg–3Li and Mg–5.5Li, however, the difference to Mg is due to the high standard deviation not significant for Mg–3Li and Mg–5.5Li (Table 2). The highest *E*_corr_ is measured for Mg–9.5Li. However, the higher potential does not directly correspond to a decrease in corrosion rate for this samples. The corrosion current densities *i*_corr_ and, thus, corrosion rates *CR*_EC_ are determined via Tafel extrapolation^51,52^ of the nearly linear branches in Fig. 5 and listed in Table 2. The corrosion rate was calculated by the following equation using the determined *i*_corr_, the molar mass *M*, Faraday constant* F*, number of electrons *n* and density *ρ*^52^:

**Table 2:** Corrosion potential *E*_corr_, corrosion current density *i*_corr_ and corrosion rate *CR*_EC_ resulting from Tafel plots obtained by potentiodynamic polarisation and corrosion rate *CR*_WL_ measured by weight loss measurements after 2 h for Mg and Mg–Li alloys (Li: 1.6% (m/m), 3% (m/m), 5.5% (m/m), 9.5% (m/m)).

	*E*_corr_ (V)	*i*_corr_ (A/m^2^)	*CR*_EC_ (mm/year)	*CR*_WL_ (mm/year)
Mg	− 1.81 ± 0.05	0.05 ± 0.01	0.12 ± 0.02	1.32 ± 0.37
Mg–1.6Li	− 1.73 ± 0.02	0.06 ± 0.02	0.13 ± 0.03	0.80 ± 0.14
Mg–3Li	− 1.76 ± 0.04	0.12 ± 0.04	0.27 ± 0.07	3.75 ± 0.68
Mg–5.5Li	− 1.77 ± 0.08	0.30 ± 0.13	0.67 ± 0.29	20.25 ± 0.33
Mg–9.5Li	− 1.63 ± 0.06	0.12 ± 0.03	0.26 ± 0.05	2.70 ± 0.38

$$\mathrm{C}{R}_{\text{EC}}=\frac{{i}_{\text{corr}}\cdot M}{\mathrm{n}\cdot F\cdot\uprho }.$$

Even though the potentiodynamic polarisation thus does not measure the steady state of the corrosion, it allows a comparison of corrosion rates determined for the different MgLi alloys. While for Mg–1.6Li the current density is similar to pure Mg, it is doubled when increasing the Li content to 3% (m/m). Mg–5.5Li exhibits the highest corrosion current density with 0.30 ± 0.13 A/m^2^. Of interest is the decrease in corrosion rate when increasing the Li mass fraction further to Mg–9.5Li which is similar to the corrosion rate of Mg–3Li.

To get further insight into the corrosion over time, weight loss studies are carried out over 2 h (Table 2). While the corrosion rate for Mg–1.6Li is lower than for pure Mg films, the same trend as for the electrochemical measurements with faster corrosion up to Mg–5.5Li and a corrosion rate of Mg–9.5Li similar to Mg–3Li is determined. Since for the weight loss measurements, the corrosion product is removed and, thus, included in the corrosion rate, this indicates that not only more material is transformed into a corrosion product layer and not released into the solution, but also that the corrosion of the material underneath is hindered. To understand the development of the corrosion rate over time, the weight loss measurements are repeated for Mg and Mg–1.6Li for 1 h and 4 h. After 1 h, the corrosion rates for both sample types are higher than after 2 h, and the difference between both sample types is even more pronounced with CR_Mg,1h_ = 2.22 ± 0.37 mm/year and CR_Mg-1.6Li,1h_ = 1.24 ± 0.15 mm/year. The corrosion rate after 4 h is similar for both materials (CR_Mg,4h_ = 0.89 ± 0.44 mm/year; CR_Mg-1.6Li,4h_ = 0.85 ± 0.27 mm/year) but lower than after 1 h and 2 h. Thus, over time the corrosion rate decreases, possibly due to passivation. It has to be noted that the difference of the rate over immersion time is also influenced by of the smaller influence of surface changes occurring due to the cleaning steps. Since the weight of the sample was limited to (3–4) mg, a slight attack of the film with the chromic acid is impacting the identified corrosion rate, especially for small overall weight changes due to short immersion times. This also leads to an increased corrosion rate measured by weight loss for thin films in comparison to larger bulk samples measured under the same conditions. The attack on Mg and Mg–1.6Li was checked to be similar after cleaning and, thus, a comparison between the thin films is possible and the faster stabilisation and passivation because of Li addition can be assumed and is not influenced by this effect.

## Discussion

The addition of Li highly influences the film growth, phases and, thus, microstructure and orientation of the Mg based thin films formed during sputtering. Changing from a strict columnar growth with a preferred (001) orientation to random orientations and the formation of Li_2_CO_3_ and oxides on the surface does not only influence the microstructure but also the resulting material properties. Thus, the properties also partially differ from results for Mg–Li bulk materials. The decrease of the tensile strength is in good agreement with literature: Mg–Li alloys show additional non-basal deformation, specifically more pronounced pyramidal and prismatic slip and twinning^46,53,54^. Because of the change in lattice structure, increasing the Li mass fraction lowers the tensile strength further. However, even though addition of Li is described to increase the maximum elongation due to the additional slip systems^53^, this is not the case for the thin films in this study. The change in microstructure and orientation and void formation for Mg–3Li and the formation of brittle carbonates (and possibly oxides) for higher Li mass fractions could influence the mechanical properties and therefore hinder the improved elongation.

### Degradation of hcp Mg–Li

For Mg–Li films in the hcp phase, an influence of the activity of lithium and the change in microstructure can influence the degradation. For the lowest Li fraction, a similar or lower corrosion rate was found during weight loss measurements in comparison to magnesium thin films prepared by sputtering with similar purity. This allows the assumption that effects on the corrosion rate are influenced by the Li content and no additional factors when comparing to bulk samples or samples with high purity. The lower corrosion rate for Mg–1.6Li at shorter times can be influenced by a faster formation of a protective corrosion product layer. Since the layer is not more stable or passivating than for Mg, the corrosion rates are converging for longer immersion times. However, this decrease does not occur for Mg–3Li. For α-phase Mg–Li alloys, Li et al. found a decrease in the corrosion rate from Mg–1Li to Mg–3Li for textured bulk materials, and therefore the opposite effect to the results presented in this study^17^. The average grain size of the investigated samples stays the same, but less groove-like corrosion and pitting occur. This is attributed to changes in the orientation and surface films^17^. Since an effect of the structure and microstructure was shown for bulk materials, it can be assumed that the change in microstructure and orientation from Mg–1.6Li to Mg–3Li also changes the properties of thin films in this study significantly. The change in structure for the thin films with an increase in grain size and a change in orientation differs from the change in structure for the bulk samples, the increase in Li included leads to an increase in corrosion rate instead of a decrease. This could also be influenced by the changes in void formation which is increased from Mg–1.6Li to Mg–3Li and, thus, might additionally increase the corrosion rate.

For Mg–3Li films, which have a random orientation of grains, the corrosion rate is higher than for Mg–1.6Li with a preferred orientation in the [001] direction. However, due to the change in Li content, the increase cannot be attributed to the orientation directly. To exclude additional influences, Mg–1.6Li films with a thickness of 20 µm and two different textures—(001) and (110) (Supplementary Fig. 2)—were prepared and analysed.

For both sample types, the texture is less preferred than for pure Mg, but either more (001) or (110) planes are aligned parallel to the surface, differing from a random orientation. Since the microstructure of both films is similar, the orientation can be assumed to be the main influencing factor. While the corrosion potential is less negative for (110) samples, those samples also exhibit a doubled corrosion current density (Table 3). Thus, the passivity is higher for (110) orientated samples, but the corrosion rate is confirmed to be slower for (001) planes. Oxidation and corrosion layers formed can strongly influence the corrosion rate, thus, the corrosion potential can give an insight into the activity of the surface. The properties of the passivating films such as density and thickness are influenced by orientation and the oxidation layers are often thinner but more stable for basal planes^38,55^, leading to a more negative potential but still decreasing possibly the corrosion rate. Processes such as pitting are found preferably on the basal planes as well^38,56^ because of a lower passivity. However, the (001) planes of the bulk material show the lowest corrosion rate according to previous studies due to the highest packing density of atoms^37,39^. Thus, the change of corrosion rate by change of orientation cannot be directly described by just taking the theoretical corrosion rates of planes into account but the orientation influences the corrosion rate of Mg–Li thin films, leading to lower corrosion rates for (001) oriented planes in this study. Therefore, it can be assumed that the increase in corrosion rate from Mg–1.6Li to Mg–3Li is influenced not only by the presence of additional Li but also the change in orientation.

**Table 3:** Corrosion potential *E*_corr_, corrosion current density *i*_corr_ and corrosion rate *CR*_EC_ resulting from Tafel plots for Mg–1.6Li films with orientations of (002) and (110).

	*E*_corr_ (V)	*i*_corr_ (A/m^2^)	*CR*_EC_ (mm/year)
Mg–1.6Li (002)	− 1.79 ± 0.03	0.08 ± 0.01	0.19 ± 0.02
Mg–1.6Li (110)	− 1.68 ± 0.04	0.16 ± 0.01	0.36 ± 0.02

### Degradation of mixed phase Mg–Li

The main effect for the highest corrosion rate being measured for Mg–5.5Li can be assigned to the inclusion of additional phases and therefore micro-galvanic coupling. The increase of the Li mass fraction and second phase should, thus, lead to an even more pronounced increase in corrosion for Mg–9.5Li. The deviation from the expected behaviour is mainly discussed to occur due to a more protective layer formed for the cubic phase which can already cover most of the surface for mixed-phase materials with a high amount of β phase and/or small grain sizes and uniform distribution^57^. The lower activity of Mg–9.5Li and increase of the corrosion potential are in good agreement with the prevention of further fast corrosion due to a passivation layer. The Li_2_CO_3_ formed in air on the surface of the film (Fig. 2) for Mg–9.5Li does not necessarily lead to a protective effect and lowering of the corrosion rate since Li_2_CO_3_ is water soluble^28^. The formation of a stable protective layer with the increase of Li content for samples in the β phase is discussed previously as the effect of the formation of Li_2_CO_3_ during corrosion or MgO doped with Li^23,26–28^. However, the formation of Li_2_CO_3_ is not specific for films with higher Li.

Since the experiments are carried out in Hanks’ balanced salt solution containing carbonates, phosphates and calcium, for all films the formation of calcium phosphates and carbonates could possibly decrease the corrosion rate. EDX studies shortly after corrosion for several days in the solution identify the corrosion products for all alloys as mainly oxygen rich, hinting at the formation of mainly oxides and hydroxides (Supplementary Fig. 3). Since the Li mass fraction is approximately 11% (m/m) in the β phase, the critical fraction of Li for doped MgO to be stable is reached (> 4.8–5.9% (m/m)) and could therefore protect the surface^28^. Further studies of the surface films and corrosion behaviour in detail are necessary to understand the corrosion process for thin films with different amounts of Li.

By comparing the different Mg–Li alloy compositions, the importance of not only the phases and microstructure but also all subsequent factors evolving by oxidation and during degradation such as protective layers are identified as the main influencing factors on the corrosion behaviour. For possible applications, the hcp phase alloys prove to be of interest due to the corrosion rate similar to pure Mg and adjustable Li release by choice of the right mass fraction of Li. Due to the better mechanical properties, Mg–1.6Li might be preferred for the use for implants if the Li release is sufficient. For treatment with a higher concentration of Li while still maintaining a relatively low degradation rate, Mg–9.5Li showed to be an interesting candidate, however, the stability over time and the influence of protective layers need to undergo further studies for reproducible use of this alloys.

## Conclusion

It was shown that freestanding Mg–Li thin films can be prepared via a combination of lithography, sacrificial layer technique and magnetron sputtering. The first studies show that the structures and properties differ depending on the Li mass fraction:

1. For hexagonal Mg–Li thin film alloys (Mg–1.6Li, Mg–3Li), the growth process and subsequent microstructure is changing from an island film growth and columnar growth to film growth and a mixed grain/columnar structure with increasing Li content. Additionally, the preferred growth with one preferred orientation for Mg is less pronounced for higher Li mass fractions.
2. For mixed-phase α + β Mg–Li thin film alloys (Mg–5.5Li, Mg–9.5Li), higher Li fractions lower the corrosion rate, possibly due to protective surface film formation. Since the thin films have a total thickness in the µm range, the formation of carbonates and oxides in air can lead to an oxidation of the whole film.
3. Even though theoretically more available gliding systems should increase the ductility for increasing Li content, this is not found for the thin film samples due to the microstructure prepared by sputtering. The tensile strength is lowered in comparison to pure Mg.
4. For Mg–1.6Li thin films, the corrosion rate is similar to Mg thin film samples. However, a faster passivation leads to a faster stabilisation of the corrosion rate over immersion time. The change in microstructure and orientation leads to increasing corrosion rates with increasing Li content for hexagonal Mg–Li. When including a second phase and therefore galvanic coupling, a high increase in the corrosion rate is found, however, the formation of protective layers for higher Li mass fractions (Mg–9.5Li) improves the corrosion resistance significantly. No final statement on the composition of the protective layers is possible with the experiments in this study.

## Materials and methods

### Film preparation

Mg–Li alloy films were prepared on silicon substrates and as freestanding thin films following the process developed by Haffner et al.^58^, consisting of a combination of UV-lithography, sacrificial layer technique with etching and sputtering. Mg–Li targets (FHR) with Li mass fractions of 2.5% (m/m), 5% (m/m), 9% (m/m) and 14% (m/m) were used. All samples were prepared in a Von Ardenne CS730S cluster machine with a base chamber pressure of < 5 × 10^–7^ mbar and 25 sccm Ar gas flow. The sputtering parameters were chosen to prepare films with low film stress at the thickness of between 10 and 20 µm and are listed in Supplementary Table 4.

A 4″ silicon (Si) wafer is coated with photoresist and structured using a mask aligner (MA6/BA6, Süss MicroTec). The structures are prepared as 15 mm × 15 mm squares for corrosion measurements and dog-bone shaped structures with a strut length of 7 mm, parallel length of 5.5 mm and a width of 0.5 mm for tensile testing. After sputtering aluminium (Al) as a hard mask, the Si wafer is etched around the Al coated structures in a deep etching step via a Bosch process (ICP-RIE SI 500, SenTech). Aluminium nitride (AlN) is deposited as a sacrificial layer before the deposition of the Mg–Li alloys. During the sputtering process, the substrate temperature was measured with temperature measuring strips. After the preparation of the final layer, Al and AlN are selectively etched in a 20%(m/m) potassium hydroxide (KOH) solution and the freestanding films are cleaned in isopropanol and distilled water. For studying the film growth of Mg–Li alloys, films on Si substrate (15 mm × 15 mm) with a thickness of 10 nm, 100 nm, 1 µm and 20 µm were deposited.

### Characterisation

The chemical composition of the samples was determined using high resolution inductively coupled plasma mass spectrometry (HR-ICP-MS, Element XR, Thermo Fisher Scientific) and atomic absorption spectroscopy (AAS, Flame AAS Agilent 240 AA, Agilent Technologies) on a minimum of three freestanding thin films per composition. For ICP-MS measurements, thin films were dissolved in ultra-pure subboiled 2%(v/v) HNO_3_. The resulting solutions were further diluted with ultra-pure DI water (MilliQ, QPod Element) at a dilution factor (DF) of 10 for the measurement of Li and Fe, and DF 2000 for the measurement of Mg. Indium (2.5 µg/L) was added to every sample solution for internal standardisation. All isotopes Li-7, Fe-56, and Mg-25 were measured in Medium Resolution mode (MR, 4000 R.P.). Accuracy of the results was monitored with certified reference materials “Trace elements in water” NIST SRM1643f and NIST SRM1640a. Measurement uncertainty as estimated from replicate analyses of sample solutions was 2–12%rel. for Li, and 0.1–9%rel. for Mg. For AAS, a 1 vol% HNO_3_ solution and dilution factors of 3–20 for Li measurements and 200 for Mg measurements were used.

The samples were additionally analysed by X-ray diffraction (Smart Lab 9 kW, Rigaku) and SEM/EDX (Ultra 55 Plus, Zeiss and ULTIM MAX 65, Oxford Instruments). The XRD scan to identify the crystallographic structure and phases was performed with a parallel beam and monochromatic Cu kα radiation on a θ/2 θ-scan with a range of 20°–90° with a speed of 5–10°/min and a step size of 0.03°. Additional reciprocal space maps were measured by combining 2D scans for sample tilt angles of χ = 0°, 15°, 30° and 45°. For studying the microstructure, cross-sections were prepared by bending the samples until fracture. SEM images are taken with an accelerating voltage of 3 kV and EDX is performed with 10 kV.

The mechanical properties were determined by uniaxial tensile testing in a BETA 5–5/6 × 10 Messphysik set-up with a strain rate of 0.4%/min. For each composition, a minimum of six dog-bone shaped samples with a thickness of approximately 20 µm were measured and the tensile strength, yield strength and elongation at fracture were compared.

### Corrosion tests

Corrosion experiments were carried out in a 155 mmol Hanks` balanced salt solution (Hanks’ balanced salts H1387, Sigma-Aldrich) with added sodium bicarbonate (0.35 g/l). For both corrosion measurements, the pH was kept around 7.4 (± 0.2) using a CO_2_ regulation and the temperature was held at approximately 37 °C. For each measurement type and composition, a minimum of three samples were tested. For electrochemical measurements, a three-electrode set-up with an Ag/AgCl reference electrode and Pt mesh counter electrode connected to a VersaSTAT 3-300 potentiostat (AMETEKSI) was used to measure linear potentiodynamic polarisation. The sample was included as the working electrode in a sample holder with an exposed area of 0.916 cm^2^. After holding the sample at the open circuit potential (*E*_OCV_) for 5 min, the measurements were carried out from − 0.3 V to + 0.3 V around *E*_OCV_ with a scan rate of 1 mV/s. Further details regarding the setup can be found in Ref.^52^. Additionally, the weight loss during 2 h of corrosion was measured by immersion tests of the samples in the solution with the same exposed area as for electrochemical measurements. The samples were cleaned from corrosion products using chromic acid solution for 15 s. Different time lengths of treatment were tested beforehand and an appropriate time for releasing the corrosion product without attacking the underlying film material in a significant amount was chosen. The sample weight was determined before exposure and after cleaning to calculate corrosion rates. For identification of corrosion products, freestanding thin films are fully immersed in the solution at RT (24 ± 2 °C) for several days without pH control and the cross-sections are analysed via EDX as described for structural measurements.

### Supplementary Information

Supplementary Information.

## Data Availability

The datasets generated during and/or analysed during the current study are available from the corresponding author on reasonable request.

## References

[1] Zhao D (2017). Current status on clinical applications of magnesium-based orthopaedic implants: A review from clinical translational perspective. Biomaterials.

[2] Moravej M, Mantovani D (2011). Biodegradable metals for cardiovascular stent application: Interests and new opportunities. Int. J. Mol. Sci..

[3] Testa L (2017). Sustained safety and clinical performance of a drug-eluting absorbable metal scaffold up to 24 months: Pooled outcomes of BIOSOLVE-II and BIOSOLVE-III. EuroIntervention.

[4] Lee J-W (2016). Long-term clinical study and multiscale analysis of in vivo biodegradation mechanism of Mg alloy. Proc. Natl. Acad. Sci. U. S. A..

[5] Campos CM (2013). Bioresorbable drug-eluting magnesium-alloy scaffold for treatment of coronary artery disease. Int. J. Mol. Sci..

[6] Zaatreh S (2017). Fast corroding, thin magnesium coating displays antibacterial effects and low cytotoxicity. Biofouling.

[7] Vallée A, Vallée J-N, Lecarpentier Y (2021). Parkinson’s disease: Potential actions of lithium by targeting the WNT/β-catenin pathway, oxidative stress, inflammation and glutamatergic pathway. Cells.

[8] Can A, Schulze TG, Gould TD (2014). Molecular actions and clinical pharmacogenetics of lithium therapy. Pharmacol. Biochem. Behav..

[9] Volkmann C, Bschor T, Köhler S (2020). Lithium treatment over the lifespan in bipolar disorders. Front. Psychiatry.

[10] Haussmann R, Noppes F, Brandt MD, Bauer M, Donix M (2021). Minireview: Lithium: A therapeutic option in Alzheimer’s disease and its prodromal stages?. Neurosci. Lett..

[11] Kirkland A, Sarlo G, Holton K (2018). The role of magnesium in neurological disorders. Nutrients.

[12] Gitlin M (2016). Lithium side effects and toxicity: Prevalence and management strategies. Int. J. Bipolar Disord..

[13] Nayeb-Hashemi AA, Clark JB, Pelton AD (1984). The Li–Mg (Lithium–Magnesium) system. Bull. Alloy Phase Diagr..

[14] Li CQ (2017). Natural ageing responses of duplex structured Mg–Li based alloys. Sci. Rep..

[15] Hsu C-C, Wang J-Y, Lee S (2008). Room temperature aging characteristic of MgLiAlZn alloy. Mater. Trans..

[16] Wang BJ, Xu DK, Cai X, Qiao YX, Sheng LY (2021). Effect of rolling ratios on the microstructural evolution and corrosion performance of an as-rolled Mg-8 wt.%Li alloy. J. Magnes. Alloys.

[17] Li C, He Y, Huang H (2021). Effect of lithium content on the mechanical and corrosion behaviors of HCP binary Mg–Li alloys. J. Magnes. Alloys.

[18] Li CQ (2018). Composition and microstructure dependent corrosion behaviour of Mg–Li alloys. Electrochim. Acta.

[19] Dobkowska A, Adamczyk-Cieslak B, Mizera J, Kubásek J, Vojtěch D (2015). Corrosion behaviour of magnesium lithium alloys in NaCl solution. Solid State Phenom..

[20] Dong L (2021). Corrosion behavior of a eutectic Mg–8Li alloy in NaCl solution. Electrochem. Commun..

[21] Wang B-J (2019). Research progress on the corrosion behavior of magnesium–lithium-based alloys: A review. Acta Metall. Sin. Engl. Lett..

[22] Taheri M, Danaie M, Kish JR (2013). TEM examination of the film formed on corroding Mg prior to breakdown. J. Electrochem. Soc..

[23] Xu W (2015). A high-specific-strength and corrosion-resistant magnesium alloy. Nat. Mater..

[24] Zeng R-C, Sun L, Zheng Y-F, Cui H-Z, Han E-H (2014). Corrosion and characterisation of dual phase Mg–Li–Ca alloy in Hank’s solution: The influence of microstructural features. Corros. Sci..

[25] Yan Y, Qiu Y, Gharbi O, Birbilis N, Nakashima PNH (2019). Characterisation of Li in the surface film of a corrosion resistant Mg–Li(-Al-Y-Zr) alloy. Appl. Surf. Sci..

[26] Hou L (2016). Investigating the passivity and dissolution of a corrosion resistant Mg-33at.%Li alloy in aqueous chloride using online ICP-MS. J. Electrochem. Soc..

[27] Chen X-B, Li C, Xu D (2018). Biodegradation of Mg–14Li alloy in simulated body fluid: A proof-of-concept study. Bioact. Mater..

[28] Yan YM (2020). On the in-situ aqueous stability of an Mg–Li–(Al–Y–Zr) alloy: Role of Li. Corros. Sci..

[29] Schlüter K (2013). Mechanical properties and corrosion behaviour of freestanding, precipitate-free magnesium WE43 thin films. Int. J. Mater. Res..

[30] Jessen LK, Zamponi C, Quandt E (2019). Mechanical properties of magnetron sputtered free standing Mg–Ag alloy films. Front. Mater..

[31] Jessen LK, Zamponi C, Willumeit-Römer R, Quandt E (2019). Magnetron sputtered freestanding MgAg films with ultra-low corrosion rate. Acta Biomater..

[32] Schlüter K, Reverey J, Hort N, Zamponi C, Quandt E (2011). Mechanical behaviour and corrosion performance of thin film magnesium WE alloys. Mater. Sci. Forum.

[33] Schlüter K (2014). Corrosion performance and mechanical properties of sputter-deposited MgY and MgGd alloys. Corros. Sci..

[34] Garcés G, Cristina MC, Torralba M, Adeva P (2000). Texture of magnesium alloy films growth by physical vapour deposition (PVD). J. Alloys Compd..

[35] Schlüter K, Zamponi C, Piorra A, Quandt E (2010). Comparison of the corrosion behaviour of bulk and thin film magnesium alloys. Corros. Sci..

[36] Blawert C (2009). Different underlying corrosion mechanism for Mg bulk alloys and Mg thin films: Different underlying corrosion mechanism for …. Plasma Process. Polym..

[37] Song G-L, Xu Z (2012). Effect of microstructure evolution on corrosion of different crystal surfaces of AZ31 Mg alloy in a chloride containing solution. Corros. Sci..

[38] Gerashi E, Alizadeh R, Langdon TG (2022). Effect of crystallographic texture and twinning on the corrosion behavior of Mg alloys: A review. J. Magnes. Alloys.

[39] Liu M, Qiu D, Zhao M-C, Song G, Atrens A (2008). The effect of crystallographic orientation on the active corrosion of pure magnesium. Scr. Mater..

[40] Wu G, Dai W, Song L, Wang A (2010). Surface microstructurization of a sputtered magnesium thin film via a solution–immersion route. Mater. Lett..

[41] Lee MH, Bae IY, Kim KJ, Moon KM, Oki T (2003). Formation mechanism of new corrosion resistance magnesium thin films by PVD method. Surf. Coat. Technol..

[42] Störmer M, Blawert C, Hagen H, Heitmann V, Dietzel W (2007). Structure and corrosion of magnetron sputtered pure Mg films on silicon substrates. Plasma Process. Polym..

[43] Messier R, Giri AP, Roy RA (1984). Revised structure zone model for thin film physical structure. J. Vac. Sci. Technol. Vac. Surf. Films.

[44] Pursel, S. M., Petrilli, J. D., Horn, M. W. & Shaw, B. A. *Effect of Alloy Addition and Growth Conditions on the Formation of Mg-Based Bioabsorbable Thin Films* (eds. Smith, G. B. & Lakhtakia, A.) 704113 (2008). 10.1117/12.796918.

[45] Blawert C (2008). Correlation between texture and corrosion properties of magnesium coatings produced by PVD. Surf. Coat. Technol..

[46] Agnew SR, Yoo MH, Tomé CN (2001). Application of texture simulation to understanding mechanical behavior of Mg and solid solution alloys containing Li or Y. Acta Mater..

[47] Atrens A (2020). Review of Mg alloy corrosion rates. J. Magnes. Alloys.

[48] Johnston S (2018). The influence of two common sterilization techniques on the corrosion of Mg and its alloys for biomedical applications: Influence of two common sterilization techniques. J. Biomed. Mater. Res. B Appl. Biomater..

[49] Johnston S (2019). Investigating Mg biocorrosion in vitro: Lessons learned and recommendations. JOM.

[50] Hofstetter J (2015). Assessing the degradation performance of ultrahigh-purity magnesium in vitro and in vivo. Corros. Sci..

[51] Geary, A. L. Electrochemical polarization. *J. Electrochem. Soc.* (1957).

[52] Jurgeleit T, Quandt E, Zamponi C (2015). Magnetron sputtering a new fabrication method of iron based biodegradable implant materials. Adv. Mater. Sci. Eng..

[53] Haferkamp, H. *et al.* Entwicklung und Eigenschaften von Magnesium‐Lithium‐Legierungen. 6 (2001).

[54] Zou Y (2016). Deformation mode transition of Mg 3Li alloy: An in situ neutron diffraction study. J. Alloys Compd..

[55] Bland LG, Gusieva K, Scully JR (2017). Effect of crystallographic orientation on the corrosion of magnesium: Comparison of film forming and bare crystal facets using electrochemical impedance and Raman spectroscopy. Electrochim. Acta.

[56] McCall CR, Hill MA, Lillard RS (2005). Crystallographic pitting in magnesium single crystals. Corros. Eng. Sci. Technol..

[57] Wang (2019). Developing improved mechanical property and corrosion resistance of Mg–9Li alloy via solid-solution treatment. Metals.

[58] Haffner, D., Zamponi, C., de Miranda, R. L. & Quandt, E. Micropatterned freestanding magnetron sputtered Mg-alloy scaffolds. *BioNanoMaterials* **16**, (2015).

