# Supplementary material for: Structural characterisation and degradation of Mg–Li thin films for biodegradable implants
Source: Sci Rep. 2023 Aug 3;13:12572. doi: 10.1038/s41598-023-39493-9 (PMC10400664; doi:10.1038/s41598-023-39493-9)
Supplement: Supplementary file 1 — Supplementary Information. [file 41598_2023_39493_MOESM1_ESM.pdf]

Structural characterisation and degradation of Mg-Li thin films for biodegradable implants - Supplementary information

Lisa Hanke[1], Lea K. Jessen[1], Felix Weisheit[1], Krathika Bhat[2], Ulrike Westernströer[3], Dieter Garbe-Schönberg[3], Regine Willumeit-Römer[2], Eckhard Quandt[1]*

[1]Inorganic Functional Materials, Institute for Materials Science, Faculty of Engineering, Kiel University, Kiel, Germany

[2]Institute of Metallic Biomaterials, Helmholtz Centre hereon, Geesthacht, Germany

[3]Marine Climate Research, Institute of Geosciences, Faculty of Mathematics and Natural Sciences, Kiel University, Kiel, Germany

Mg-Li alloy	Li (%(m/m))			Fe (%(m/m))
	Target	Film, ICP-MS	Film, AAS	Film, ICP-MS
Mg-1.6Li	2.5	1.60±0.06	1.55±0.03	0.0029±0.0008
Mg-3Li	5	3.07±0.12	3.13±0.08	0.0027±0.0003
Mg-5.5Li	9	5.15±0.69	6.12±0.32	0.0028±0.0004
Mg-9.5Li	14	9.31±0.84	9.88±0.07	0.0033±0.0006

Table 1 Mass fractions of Li in targets (nominal) and prepared freestanding thin films analysed by ICP-MS and AAS. Additionally, the Fe contamination measured by ICP-MS is given. The measurement uncertainties given are statistical deviations from minimum 3 samples.

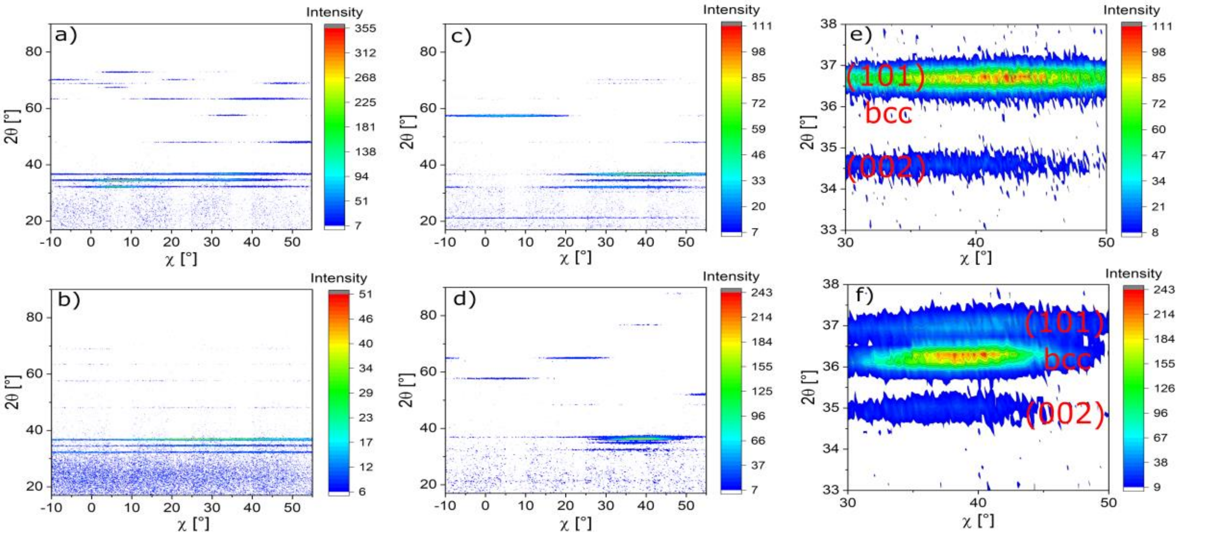

Figure 1 Reciprocal space maps measured with 2D- detector and sample tilting to χ=0°,15°,30°,45° for a) Mg-1.6Li b) Mg-3Li c) Mg-5.5Li d) Mg-9.5Li and zoomed in area around the (002) and (101) hcp and (110) bcc peak for e) Mg-5.5Li and f) Mg-9.5Li.

	Mg-1.6Li	Mg-3Li	Mg-5.5Li	Mg-9.5Li
a [Å]	3.208±0.002	3.202±0.002	3.196±0.004	3.200±0.002
c [Å]	5.197±0.005	5.175±0.007	5.156±0.010	5.161±0.008

Table 2 Average lattice constants *a* und *c* and standard deviations calculated from XRD results for Mg-Li alloys (Li: 1.6 %(m/m), 3 %(m/m), 5.5 %(m/m), 9.5 %(m/m)).

	Mg-1.6Li	Mg-3Li	Mg-5.5Li	Mg-9.5Li
hcp				
100	32.16±0.03	32.25±0.03	32.35±0.08	32.27±0.06
002	34.48±0.03	34.67±0.06	34.78±0.07	34.75±0.07
101	36.63±0.03	36.75±0.03	36.87±0.08	36.78±0.05
102	47.90±0.02	48.09±0.03	48.28±0.10	48.21±0.04
110	57.44±0.01	57.55±0.03	57.69±0.09	57.62±0.05
103	63.26±0.02	63.51±0.04	63.79±0.09	63.75±0.05
200	67.40±0.02	67.56±0.03	67.71±0.07	67.57±0.01
112	68.79±0.02	69.00±0.02	69.16±0.09	69.11±0.06
201	70.11±0.02	70.28±0.04	70.42±0.10	70.33±0.03
004	72.85±0.07	73.21±0.09	73.45	73.48±0.01
202		78.24±0.05		78.33±0.01
104	81.90±0.04	82.23±0.06	82.64±0.12	82.65±0.10
bcc				
110			36.10±0.07	36.03±0.06
200			51.66±0.10	51.48
211			64.89±0.06	64.84±0.07
Li_2CO_3				
110			21.39±0.09	21.29±0.06
200			23.38±0.06	23.32±0.07
$11\bar{1}$			23.49	
111			29.48±0.04	29.38±0.03
$20\bar{2}$			30.67±0.09	30.52±0.06
002			31.77±0.06	31.65±0.05
$11\bar{2}$			34.11±0.01	34.02±0.06
020			36.13±0.03	36.06±0.03
021			39.65±0.01	39.64±0.10
310			39.93±0.02	39.91
$22\bar{1}$				42.60
112				42.60
220			43.55±0.02	43.45
130				56.77±0.04
$33\bar{1}$				65.41

Table 3 Average 2θ angles (°) from XRD diffractograms for Mg-1.6Li, Mg-3Li, Mg-5.5Li and Mg-9.5Li with corresponding miller indices for hcp and bcc Mg-Li and Li_2CO_3 with. A minimum of three samples was measured and standard deviations are given if the peak was present in multiple diffractograms.

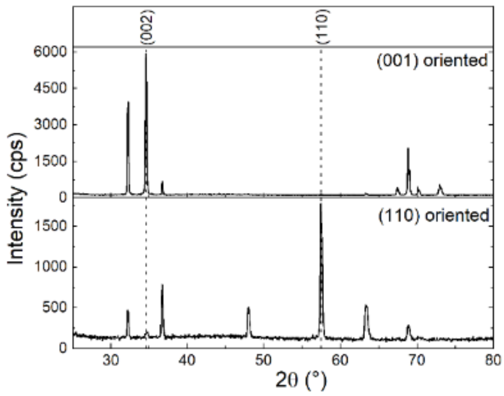

Figure 2 XRD diffractograms for Mg-1.6Li thin films with orientations of (002) and (110). The main orientations are indicated.

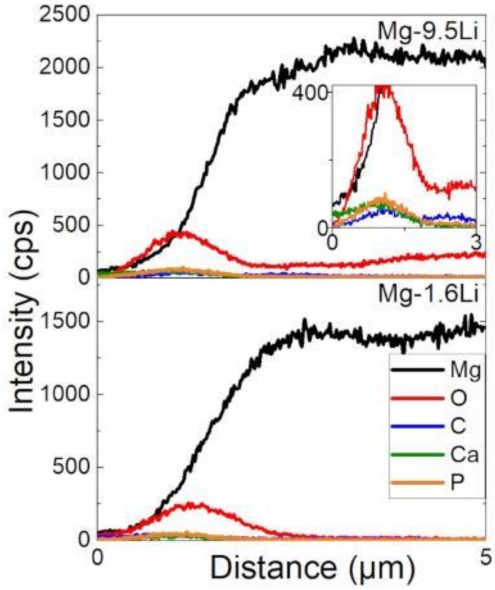

Figure 3 EDX line scans of cross sections of Mg-1.6Li and Mg-9.5Li thin films after corrosion in HBSS over 3-5 days. The inset shows the area of the corrosion layer.

MgLi alloy	Power (W)	Pressure (10^{-3} mbar)	Sputtering rate (nm/s)
Mg-1.6Li	50	2.3	1.45
Mg-3Li	50	3.3	1.75
Mg-5.5Li	50	2.5	1.17
Mg-9.5Li	50	2.3	0.88

Table 4 Sputter parameters for Mg-Li sputtering at standard parameters for stress-free freestanding thin films and for Mg-3Li at different pressure and power.
